# Optimization of Pectinase-Assisted Extraction from Date Palm and Development of a Bael–Jujube Ready-to-Drink Beverage: A Two-Stage Approach

**DOI:** 10.3390/foods15081394

**Published:** 2026-04-16

**Authors:** Saeid Jafari, Pitchaya Tuntiteeraboon, Isaya Kijpatanasilp, Sochannet Chheng, Kuan-Chen Cheng, Kitipong Assatarakul

**Affiliations:** 1 Department of Food Technology, Faculty of Science, Chulalongkorn University, Bangkok 10330, Thailand; saeid.j@chula.ac.th (S.J.); ykijpat@gmail.com (I.K.); sochannetc@gmail.com (S.C.); 2 Department of Food Chemical Engineering, Kampong Speu Institute of Technology, Chbar Mon 050601, Cambodia; 3 Institute of Food Science and Technology, National Taiwan University, No. 1, Section 4, Roosevelt Rd., Taipei 106216, Taiwan; kccheng@ntu.edu.tw; 4 Institute of Biotechnology, National Taiwan University, No. 1, Section 4, Roosevelt Rd., Taipei 106216, Taiwan; 5 Department of Optometry, Asia University, No. 500, Lioufeng Rd., Wufeng, Taichung 413305, Taiwan; 6 Department of Medical Research, China Medical University Hospital, China Medical University, No. 91, Hsueh-Shih Road, Taichung 404333, Taiwan; 7 Department of Food Science, Fu Jen Catholic University, No. 510, Zhongzheng Rd., New Taipei City 242062, Taiwan

**Keywords:** characteristics, date palm, enzyme treatment, extraction, functional beverage, sensory analysis

## Abstract

Rising consumer demand for functional beverages has accelerated the development of health-promoting, fruit-based ready-to-drink (RTD) products. This study investigated the effects of incubation temperature (50–80 °C) and time (60–240 min) on pectinase-assisted extraction (0.1% *v*/*v*) of date palm (*Phoenix dactylifera* L., Bahi variety) juice and subsequently formulated antioxidant-rich RTD beverages by blending the optimized extract with bael and jujube juices. The optimal extraction condition (50 °C, 60 min) was selected based on maximizing bioactive compound recovery rather than yield, achieving total phenolic content of 326.33 mg GAE/100 mL, total carotenoid content of 1.08 mg β-carotene equivalents/100 mL, and strong antioxidant activity (DPPH: 514.06; FRAP: 595.38 µmol TE/100 mL). Although maximum yield (81.25%) was obtained at 60 °C for 240 min, functional quality was prioritized. Six RTD formulations were developed using a constrained simplex-lattice mixture design. All blends exhibited significantly enhanced phenolic content, carotenoids, and antioxidant capacity compared to the control, while pH and acidity remained stable (*p* > 0.05). Sensory evaluation indicated that the formulation containing 70% date palm, 15% bael, and 15% jujube achieved the highest acceptability (6.50). These findings highlight the potential of this tri-fruit blend as a functional RTD beverage, warranting further studies on shelf-life stability.

## 1. Introduction

In recent years, the demand for value-added and/or functional food items has exploded. Fruits and fruit derivatives (such as beverages) are excellent sources of bioactive chemicals, which have a wide range of health benefits. The date palm (*Phoenix dactylifera* L.) is a source of carbohydrates, dietary fiber, vitamins (C, B1, B2, A, riboflavin, and niacin), and minerals that are prevalent in Middle Eastern countries (calcium, iron, copper, magnesium, manganese, phosphorus, potassium, sodium and zinc) [1]. It is a phytochemical-rich product that contains phenolic, carotenoid, and tannins, in addition to its nutritional value [2].

The date palm can be processed into a variety of items, particularly date palm juice products. The extraction of juice from the fruits of date palms, where the date palm fruit includes pectin that connects the cells of the tissue, giving the date palm fruit a firm texture, is known as date palm juice processing [3]. Thus, the use of pectinase enzymes, which catalyze the cutting of bonds in the pectin compound frame on the walls of plant cells, causes a loss of texture stabilization characteristics, resulting in an increase in the number of released substances such as pigments, antioxidants, and flavoring agents [4]. Dwivedi et al. [5] reported that enzymatic extraction is a commonly applied and straightforward approach for recovering phenolic compounds from dates, as it enhances their release under gentle conditions by degrading cell wall structures. This method generally offers higher yields, improved sustainability, and stronger selectivity, making it highly suitable for use in food and nutraceutical applications.

Researchers have increasingly focused on the development of healthier beverage formulations [6,7,8]. As a result, a ready-to-drink beverage containing date palm extract and two other herbs, bael and jujube juices, may be a viable choice. Bael and jujube juices are both recognized for their rich composition of bioactive compounds and associated health-promoting properties. Bael juice contains carotenoids, phenolics, alkaloids, pectins, tannins, coumarins, flavonoids and terpenoids. In the Ayurvedic system of medicine, it can be use to aid digestion and stomach irritation and prevent sub-acute and chronic dysentery due to its biological properties [9]. In contrast, jujube juice is especially rich in vitamin C, flavonoids, polysaccharides, and triterpenic acids, which confer potent antioxidant activity, immune enhancement, hepatoprotective effects, and notable neuroprotective and anti-stress functions [10]. While both juices support overall health through their antioxidant and antimicrobial activities, bael juice is particularly linked to gastrointestinal and metabolic benefits, whereas jujube juice is more associated with immune modulation, stress reduction, and liver protection. Together, these fruits represent valuable functional ingredients with broad potential applications in health-promoting beverages.

In this study, date palm was selected as the base matrix due to its high natural sugar content (primarily glucose and fructose), abundant pectin structure (ideal for enzymatic clarification), and established nutritional profile rich in minerals and phenolics [1,2]. Its mild, caramel-like sweetness provides a versatile sensory foundation that masks potential astringency from polyphenol-rich additives. Bael and jujube were strategically chosen to complement this base: bael contributes moderate acidity and is a good source of pectin; it also contains phenolic compounds, including chlorogenic acid, which contribute to its antioxidant capacity without imparting excessive tartness, while jujube introduces unique neuroprotective flavonoids (spinosin, swertisin) and a subtle, floral-sweet aroma that rounds out the flavor profile [9]. Together, these three fruits create a phytochemically diverse, sensorially balanced matrix suitable for functional beverage development [6].

Despite the known health benefits of bael and jujube, there is a lack of research on their combined use in a ready-to-drink functional beverage produced via pectinase-assisted extraction of date palm. Specifically, the trade-off between extraction yield and bioactive retention in date palm matrices remains underexplored, as does the application of mixture design to link composition to sensory and functional properties in this specific tri-fruit blend. As a result, the goal of this study was to investigate the effects of temperature and incubation time on pectinase-assisted extraction (0.1% *v*/*v* enzyme) of date palm juice and to determine the condition that maximizes bioactive compound recovery. Furthermore, a date palm juice-based ready-to-drink beverage was described in terms of physical-chemical characteristics and sensory evaluations.

## 2. Materials and Methods

### 2.1. Chemicals

All chemicals used were of analytical grade. The following reagents were obtained from commercial sources with specified purities: 2,2-diphenyl-1-picrylhydrazyl (DPPH, ≥97% purity, Sigma Aldrich, St. Louis, MO, USA); 6-hydroxy-2,5,7,8-tetramethylchromane-2-carboxylic acid (Trolox, ≥98% purity, Sigma Aldrich, USA); β-Carotene (≥95% purity, type II synthetic, Sigma Aldrich, USA); Citric acid (C_6_H_8_O_7_, food grade, ≥99.5%, Union Chemical 1986, Nonthaburi, Thailand); Folin–Ciocalteu reagent (2N, Loba Chemie, Mumbai, India); Ferric chloride (FeCl_3_·6H_2_O, ≥99%, POCH S.A., Gliwice, Poland); Gallic acid (≥98% purity, Sigma Aldrich, USA); Tripyridyltriazine (TPTZ, ≥99% purity, Sigma Aldrich, USA). Pectinase from *Aspergillus niger* (EC 3.2.1.15, 60,000 IU/mL, iKnowZyme^®^, Reach Biotechnology, Pathum Thani, Thailand) was used as received without further purification.

### 2.2. Sample Preparation

Fresh date palms (Bahi variety) at the rutab stage (moisture content 45–50%, total soluble solids 30–35 °Brix, confirmed by refractometer upon receipt) were delivered from Kanchanaburi province, Thailand. Yaowarat Old Market in Bangkok, Thailand, also provided dried Bael and Jujube. All samples were delivered to Chulalongkorn University’s Department of Food Technology. The date palms were dried at 60 °C (moisture 5%) in a hot air oven (Memmert, DO 6062, Schwabach, Germany) to ensure microbial stability during transport/storage and standardize solid content for reproducible extraction ratios, vacuum-packed in an aluminum-laminated foil bag, and stored at −20 °C for further use.

### 2.3. Pectinase-Assisted Extraction of Date Palm

After removing the seed, 25 g of fresh date palm fruit (pre-drying weight basis) was sliced into small pieces and blended with 75 mL of distilled water (date palm: distilled water 1:3 *w*/*v*) (Waring Commercial, model 8010BU, Stamford, CT, USA). Pectinase concentration was fixed at 0.1% (*v*/*v*) (equivalent to approximately 60 IU/g fruit), based on our previous study [4] and supplier recommendations for fruit juice clarification. Temperature and incubation time were varied to determine their effects on extraction efficiency and bioactive compound recovery. Pectinase content (0% for the control sample and 0.1% *v*/*v*), temperature (50, 60, 70, and 80 °C), and time (60, 120, 180 and 240 min) were the extraction variables. The samples were adjusted to pH 4.0 with 1% (*w*/*v*) citric acid solution before adding the enzyme (0.1% *v*/*v*). Pectinase from *Aspergillus niger* (EC 3.2.1.15, iKnowZyme^®^, Reach Biotechnology, Thailand) with declared activity of 60,000 IU/mL; optimum pH 3.5–5.0, temperature 40–60 °C per datasheet; contained polygalacturonase as main activity with minor cellulase/hemicellulase side activities. The samples (control and pectinase-assisted extracts) were centrifuged at 3000 rpm (approx. 1500× *g*, rotor radius 15 cm) for 10 min and filtered using Whatman paper No. 4 after extraction. For later usage, the filtered samples were maintained at 4 °C. Controls underwent identical pH, temperature, and time incubation without enzyme addition (Table 1).

### 2.4. Physical Characteristics

#### 2.4.1. Yield (%)

The yield (%) was calculated by measuring the volume of date palm juice before pectinase extraction and the volume of date palm juice after pectinase extraction using the following equation:

$$Yield(\%)=\frac{Volume\;of\;extracted\;date\;palm\;juice}{Initial\;volume\;of\;date\;palm\;juice}\times \;100$$

#### 2.4.2. Color

The color was measured using a Chroma meter (Monica Minolta CR-400, Tokyo, Japan), the CIE color system, and the machine adjusted the L* a* and b* values before each sample measurement. L* stands for lightness, with values ranging from 0 (dark) to 100 (white), a* for redness (+a*) and greenness (−a*), and b* for yellowness (+b*) and blueness (−b*). The color difference (E*) was calculated using the following formula:

∆E* = [(L*_1_ − L*_2_)^2^ + (a*_1_ − a*_2_)^2^ + (b*_1_ − b*_2_)^2^]^1/2^

where subscript 1 is the default color value of the sample and subscript 2 is the color value measured at a time of the sample.

### 2.5. Chemical Analyses

Total soluble solid content (°Brix) was measured by a digital refractometer (HI96801, Hanna, Bangkok, Thailand) at 0–85 °Brix. The pH of the date slurry was adjusted to 4.0 ± 0.1 using a 1% (*w*/*v*) citric acid solution (prepared by dissolving 10 g citric acid in 1 L distilled water). pH was verified after enzyme addition using a calibrated pH meter (Mettler Toledo, Greifensee, Switzerland); no further adjustment was needed as the enzyme solution itself had pH 4.2. The determination of total acid content (% citric acid) was conducted [11].

$$Total\;acid\;content(\%\;citric\;acid)=\frac{\mathrm{Titer}\;\times \;N\;\times \;n\;\times \;100}{\mathrm{Sample}\;\mathrm{volume}}$$

where “N” is the concentration of sodium hydroxide (normal) and “n” is the milliequivalent weight of citric acid, equal to 0.07.

TPC was determined using the Folin–Ciocalteu method: 100 μL sample was mixed with 7 mL distilled water and 500 μL Folin–Ciocalteu reagent, incubated for 5 min, then 400 μL 7.5% (*w*/*v*) sodium carbonate was added. After 30 min of dark incubation, absorbance was measured at 765 nm. Calibration used gallic acid standards (0–500 mg/L, R^2^ = 0.999). Results were not corrected for reducing sugars; however, preliminary tests with sugar-matched blanks (glucose + fructose at concentrations equivalent to sample °Brix) showed minimal interference (<3% of measured absorbance), consistent with previous reports for date matrices.

For TCC, samples were extracted with hexane: acetone: ethanol (2:1:1, *v*/*v*/*v*) according to Oo et al. [12]. The organic phase was collected and the absorbance was measured at 450 nm. β-carotene standards (0–10 μg/mL in hexane, Sigma-Aldrich, purity ≥ 95%) were used for calibration (R^2^ = 0.998). Results were expressed as mg β-carotene equivalents/100 mL using the extinction coefficient E^1^% = 2590 at 450 nm in hexane.

The antioxidant activity was measured using the 2,2-diphenyl-1-picrylhydrazyl (DPPH) and ferric reducing antioxidant power (FRAP) techniques, as described by Jafari et al. [13]. For DPPH assay: 250 μL sample was mixed with 4.75 mL DPPH solution (0.1 mM in methanol). After 30 min dark incubation at 25 °C, absorbance was measured at 515 nm. Trolox standards (0–500 μM) were used for calibration. For FRAP assay: FRAP reagent was prepared fresh by mixing with 300 mM acetate buffer (pH 3.6), 10 mM TPTZ in 40 mM HCl, and 20 mM FeCl_3_·6H_2_O (10:1:1 *v*/*v*/*v*). Sample (50 μL) was mixed with 950 μL FRAP reagent, incubated 4 min at 37 °C, and absorbance read at 593 nm. Both assays used appropriate dilution factors to ensure measurements fell within the linear range of standard curves.

### 2.6. Developing Ready-to-Drink Beverage Formulations from Optimized Date Palm Juice Extract

The blending proportions for the ready-to-drink (RTD) beverages were designed using a constrained simplex-lattice mixture design ({3,2} order) with three components (date palm extract, bael juice, and jujube juice) that sum to 100%. The experimental region was restricted to formulations containing 50–70% date palm extract, 15–25% bael juice, and 15–25% jujube juice (*v*/*v*). This resulted in six formulations: five experimental blends and one control (100% date palm extract). The exact proportions of each formulation are presented in Table 2. All formulations were prepared in triplicate (*n* = 3) for physical-chemical and functional analyses. Dry bael (10 g) was mixed with 100 mL of drinking water for this experiment (dry bael proportion: water = 1:10 *w*/*v*). In addition, 10 g of dried jujube was mixed with 100 mL of drinking water (dry jujube proportion: water 1:10 *w*/*v*). After that, each was cooked for 10 min at 100 °C before being filtered. To make a ready-to-drink beverage formula, bael and jujube juices were combined with date palm extract in differing proportions (Table 2). The control sample was made up entirely of date palm extract. The physical-chemical properties of ready-to-drink beverages were determined in the same way as in the preceding section.

### 2.7. Sensory Analysis

Sensory evaluation was performed in a single session with 30 untrained panelists (15 male, 15 female; 20–25 years old), recruited from university staff and students. The study was approved by the Chulalongkorn University Ethics Committee, and informed consent was obtained. Each panelist evaluated all six formulations in a randomized order using a 9-point hedonic scale to determine their acceptance of color, scent, flavor, and overall preference. Samples (30 mL) were served at 10 °C in coded plastic cups. Sensory evaluation was performed using a structured 9-point hedonic scale (1 = dislike extremely, 9 = like extremely) with explicit attribute anchors to standardize panelist interpretation: Color (clarity, hue intensity, absence of undesirable browning), Smell/Aroma (fresh fruit intensity, presence of cooked/fermented off-notes, balance of floral/citrus notes), Sweetness (perceived sugar intensity, cloying vs. balanced), Sour taste (acid sharpness, astringency from polyphenols), Flavor (overall taste harmony, aftertaste length, fruit authenticity), and Overall preference (willingness to consume/purchase). Panelists were instructed to rinse with water and unsalted crackers between samples. Future work will incorporate trained sensory panels and Quantitative Descriptive Analysis (QDA) to map specific volatile and taste-active compounds to consumer perception.

### 2.8. Statistical Analysis

The extraction study was conducted using a 2 × 4 × 4 factorial arrangement (enzyme concentration × temperature × extraction time) within a completely randomized design (CRD), with four analytical replicates for each treatment. The influence of enzyme level, temperature, and extraction time on the measured parameters was assessed using analysis of variance (ANOVA), focusing on the main effects of each factor, while interaction effects were not evaluated. For the ready-to-drink beverage formulations, a constrained mixture design consisting of six formulations (Table 2) was implemented. Each formulation was prepared in three independent production batches (n = 3), and all physicochemical and functional measurements were performed in duplicate for each batch. Sensory evaluation was carried out with 30 panelists. Statistical analysis of both the extraction experiment and the beverage formulation data (physicochemical, functional, and sensory attributes) was performed using one-way ANOVA, followed by Tukey’s HSD test for mean comparison at a significance level of *p* ≤ 0.05. All analyses were conducted using IBM SPSS Statistics version 22. Prior to analysis, the assumptions of normality and homogeneity of variance were verified. Results are presented as mean ± standard deviation.

## 3. Results and Discussion

### 3.1. Effect of Temperature and Incubation Time on Pectinase-Assisted Extraction of Date Palm Juice

Table 3 indicates the impact of enzyme-assisted extraction settings on date palm juice extraction yield. The extraction yield was found to be impacted by enzyme inclusion, temperature, and time (*p* < 0.05). For control samples (no enzyme) and pectinase-assisted extracts of date palm, yields varied from 54.60 to 70.14% and 63.22 to 81.25%, respectively. Temperature (60 °C) and time (240 min) were shown to be the most effective extraction conditions for pectinase-assisted date palm extracts (81.25%). Tuntiteeraboon et al. [4] revealed that pectinase may degrade pectin in the initial cell wall and the welded wall between cells, resulting in a stronger effect and the extraction of a greater number of essential chemicals. As previously stated, both temperature and time had a substantial impact on the extraction yield, which might be explained by increasing enzyme hydrolysis and polysaccharide breakdown in pectin at higher temperatures (60 °C) and longer times (240 min).

The inclusion of temperatures (70–80 °C) exceeding the enzyme’s optimal range (45–55 °C per manufacturer) was intentional to distinguish between enzymatic and thermal effects on extraction. At these elevated temperatures, pectinase is expected to undergo rapid thermal denaturation (confirmed by preliminary activity assays showing <10% residual activity after 30 min at 70 °C). Therefore, any extraction enhancements observed at 70–80 °C can be attributed primarily to thermal softening and hydrolysis rather than enzymatic action. This experimental design allows assessment of the relative contributions of enzymatic vs. thermal mechanisms across the full temperature spectrum, with the 50–60 °C range representing conditions where enzymatic activity predominates. An increasing extraction’s yield (83.40%) was reported for pectinase-assisted (0.1%) extracts of cashew juice at 48 °C [14]. The extraction conditions at that study were temperature (30–50 °C), time (20–60 min) and the concentration of pectinase was 0.08–0.1%. Khandare et al. [15] also confirmed that using pectinase (0.2%) improved the extraction yield (64%) of purple carrot juice extracted at 60 °C for 60 min. Although the highest juice yield (81.25%) was obtained at 60 °C and 240 min (Table 3), this decision for the optimum condition was based on prioritizing the recovery of bioactive compounds. Prolonged incubation and higher temperatures likely caused partial degradation of heat-sensitive phenolics and carotenoids, outweighing the marginal gain in yield. This trade-off is consistent with previous enzyme-assisted extraction studies that prioritize functional properties over maximum yield when developing antioxidant-rich beverages.

### 3.2. Characterization

Total soluble solids (TSS) are commonly used as an indicator of concentration in liquid foods. In fruit and vegetable juices, TSS primarily reflects the concentration of soluble components—mainly sugars (such as sucrose, glucose, and fructose), along with organic acids and other minor soluble constituents. It can be used as an index to measure the aging of fruits and vegetables. Our current results showed that pectinase-assisted extracts of date palm juice had no effects on the total soluble solids (*p* > 0.05), ranging from 6.4 to 6.8 °Brix (Table 4). The maximum numerical value was obtained when date palm juice was extracted with pectinase and incubated at 60 °C for 240 min. The effect of pectinase on the total soluble solids in blueberry extract was investigated by Siddiq et al. [16], who discovered that utilizing the enzyme had no influence on the total soluble solids, which ranged from 7.41 to 7.44 °Brix. Norjana and Noor Aziah [17], on the other hand, found that increasing pectinase concentration increased the total soluble solids in durian juice from 6.50 to 9.00 °Brix.

Food color is a visible physical feature that influences customer satisfaction. The L* value (which indicates the sample’s brightness; the greater the value, the brighter the sample) varied from 78.78 to 94.58 among the samples (Table 5). Due to the browning reaction, increasing the extraction temperature from 50 to 80 °C decreased the L* value (*p* ≤ 0.05). The L* value in the pectinase-assisted extracts was higher than in the control samples, possibly because pectinase allowed protein molecules in colloids and pectin to merge into big and dense molecules, making the juice clearer [18]. Norjana and Noor Aziah [17] discovered that after 3 h of extraction at 38.5 °C, the L* value of pectinase-assisted (0.075%) extracts of durian juice declined. Lee and Coates [19] also discovered that grapefruit pasteurization techniques resulted in a decreased L* value, explaining that thermal processing caused some particles in the juice to precipitate, resulting in those results. As shown in Table 5, the a* value ranged from −14.320.40 to −21.490.04, with negative values indicating that the samples were green in color. The b* value was also in the 48.23 to 84.98 range (b* positive implies yellow, and b* negative means blue). Furthermore, ∆E*, which denotes a color difference, ranged from 0.20 to 0.40. The highest ∆E* was recorded for samples extracted at 60 °C.

Gani et al. [20] identified 1.90% enzyme concentration, 30 °C, and 120 min as the optimal conditions for pectinase-assisted extraction of pear juice from the William Bartlett variety. The variation between their optimum and the conditions established in our study (50 °C for 60 min) can be explained by differences in fruit structure and extraction aims. Dates possess higher amounts of fiber, pectin, and strongly bound phenolic compounds, which necessitate a moderately higher temperature to activate pectinase efficiently and facilitate cell wall breakdown. Pears, by contrast, have a softer texture and less tightly bound phenolics, enabling effective extraction at lower temperatures. Additionally, moderate heat in our process did not negatively impact date bioactives, whereas the pear study noted reductions in ascorbic acid and phenolics at higher temperatures, supporting their choice of a milder thermal setting. The reduced extraction time in our study also indicates quicker enzymatic action on the denser date matrix at 50 °C, while pears required prolonged incubation to achieve desirable clarity and yield. Overall, the 50 °C for 60 min condition in our research reflects a more thermally driven and rapid extraction suited to the structural characteristics of date palm, whereas the pear study’s lower temperature and longer duration align with its softer tissue properties and differing optimization objectives.

### 3.3. Functional Properties of Optimized Date Palm Extract

Table 6 shows how temperature and time affected the TPC value, which ranged from 134.67 to 326.33 mg GAE/100 mL (*p* ≤ 0.05). The pectinase-assisted extracts had a higher TPC value than the control sample at 60 min (326.33 vs. 313.00 mg GAE/100 mL, respectively). The greater TPC value was due to pectinase’s ability to break down the fruit’s cell walls, allowing more phenolic chemicals to be released.

Khandare et al. [15] also discovered that extracting purple carrot juice with pectinase increased the amount of TPC and anthocyanins. In our investigation, however, increasing the temperature and time of extraction reduced the TPC value. These findings suggest that temperature and time may have an impact on the TPC value. Marathe et al. [21] discovered that factors affecting enzyme function, such as increasing enzyme concentration, temperature, and time, alter bioactive substance extraction. The decrease at higher temperatures is likely due to thermal degradation and oxidation of phenolic compounds.

The date palm contained carotenoids classified as β-carotene and lycopene, according to a prior study [22]. Table 6 shows that the TCC values in the current investigation varied from 0.33 to 1.08 mg β-carotene equivalents/100 mL. At 50 °C, pectinase-assisted extracts had the highest (*p* ≤ 0.05) TCC value. Consuming foods high in carotenoids can prevent the degeneration of cells in the body and minimize the chance of illness development. Carotenoids are antioxidants that protect cells and tissues from harm caused by free radicals. The TCC of samples extracted with pectinase increased by 31.25%. Increasing the temperature to 70 and 80 °C and lengthening the incubation time resulted in a lower TCC value, which was consistent with Asaduzzaman’s [23] findings. This reduction is attributed to thermal isomerization and oxidation of carotenoids.

The samples had DPPH values ranging from 243.23 to 514.06 µmol TE/100 mL, with pectinase-assisted extracts at 60 °C having the highest DPPH (514.06 µmol TE/100 mL) (Table 6). Increasing the temperature and time of extraction, like with TPC and TCC, resulted in a decreased DPPH value. Wongsariya and Kanchanadumkerng [24] discovered that when longan was extracted using pectinase (0.5–3.0%) for 0.5 to 5 h, the DPPH value was highest. Their findings were linked to pectinase’s hydrolyzing actions on cell walls, which resulted in the release of more antioxidants, as well as the fact that utilizing pectinase boosted the efficiency of extracting biomaterials, as previously discussed. Another method for determining the antioxidant properties of plant materials is FRAP. It is based on purple-blue redox reactions (Fe^3+^-TPTZ→ Fe^2+^ TPTZ). Table 6 shows that the FRAP values ranged from 240.98 to 595.38 µmol TE/100 mL. At 60 °C, pectinase-assisted extracts with 595.38 µmol TE/100 mL had the highest FRAP value (*p* ≤ 0.05).

Overall, the decrease in TPC, TCC, and antioxidant activity at higher temperatures (70–80 °C) and prolonged times (180–240 min) can be attributed to multiple mechanisms: (1) Thermal degradation of heat-labile phenolic compounds, particularly phenolic acids susceptible to decarboxylation above 60 °C; (2) Oxidative polymerization of phenolics, forming high molecular weight complexes with reduced Folin–Ciocalteu reactivity; (3) Isomerization and degradation of carotenoids, which are highly susceptible to thermal cis-trans isomerization and oxidation at elevated temperatures; (4) Maillard reaction products at higher temperatures may interfere with antioxidant assays, though their contribution is likely minimal under these conditions; (5) Potential enzyme inactivation beyond 60 °C, limiting continued release of bound bioactives. The consistent pattern across all bioactive markers (TPC, TCC, DPPH, FRAP) supports thermal degradation as the dominant mechanism.

### 3.4. Ready-to-Drink Beverage Formulations from Date Palm Juice

#### 3.4.1. Characteristics

The growing consumer awareness of health and wellness has driven a significant surge in demand for functional beverages, particularly those derived from fruit-based ingredients [6,25]. This trend aligns with the objective of this study: to develop a novel, palatable, and commercially viable ready-to-drink (RTD) beverage by synergistically combining pectinase-optimized date palm extract with bael and jujube juices. Utilizing a constrained mixture design, five distinct formulations were created (Table 2), blending date palm extract (50–70%) with bael juice (15–25%) and jujube juice (15–25%), alongside a control of 100% date palm extract.

The physicochemical stability of RTD beverages is critical for product quality and shelf life. Acidity, which influences flavor profile, microbial stability, and overall sensory perception, remained consistent across all formulations. No significant differences (*p* > 0.05) were observed in pH (4.49–4.56) or titratable acidity (0.14–0.15% citric acid) (Table 7). This stability is advantageous for industrial production, suggesting that blending these fruit juices does not introduce significant acid-base variations that would require major adjustments. In contrast, the total soluble solids (°Brix) exhibited significant variation (*p* ≤ 0.05), ranging from 6.2°Brix in Formulation 4 to 8.0 °Brix in the control. The lower °Brix in the blends can be attributed to the dilution effect from bael and jujube infusions, which were prepared at a lower solids concentration (1:10 *w*/*v* fruit-to-water) compared to the concentrated date palm extract. Similar dilution effects on total soluble solids have been reported in blended juices, where the addition of less concentrated components modulates the final °Brix [26].

Color is a primary attribute influencing consumer purchase decisions. The CIELAB color values (L*, a*, b*, ∆E) of the RTD formulations differed significantly (*p* ≤ 0.05) from the control (Table 7). The control sample (100% date palm extract) exhibited the highest lightness (L* = 93.56) and yellowness (b* = 75.24), characteristic of the amber hue of date juice. The blended formulations showed substantially lower L* and b* values, resulting in a darker, less yellow, and more reddish-brown appearance, as indicated by higher positive a* values (12.33–19.00). This pronounced color shift (∆E* up to 69.97) is a direct consequence of blending with deeply pigmented bael and jujube juices, both known for their rich polyphenolic and carotenoid content, which contribute to darker hues [9,10]. The significant ∆E* values confirm that the blends are visually distinct from plain date palm juice, offering a unique color profile. Similar marked color changes have been documented in other multi-fruit juice blends, where the combination of pigments from different sources creates a new composite color [27].

**Table 7:** Effects of optimized pectinase-assisted extraction of date palm on physical-chemical characteristics of Ready-to-Drink beverages.

	Treatments (Date Palm Juice Extract: Bael Juice: Jujube Juice)					
	1	2	3	4	5	6
Total acid content (%citric acid) ^ns^	0.14 ± 0.03	0.15 ± 0.02	0.14 ± 0.02	0.14 ± 0.02	0.14 ± 0.02	0.14 ± 0.06
pH ^ns^	4.51 ± 0.32	4.56 ± 0.28	4.49 ± 0.33	4.53 ± 0.30	4.53 ± 0.30	4.52 ± 0.31
Total soluble solids (°Brix)	6.9 ± 0.45 ^b^	6.4 ± 0.80 ^b^	6.3 ± 0.87 ^b^	6.2 ± 0.97 ^b^	6.6 ± 0.71 ^b^	8.0 ± 0.33 a
Color values						
L*	53.33 ± 4.64 ^b^	46.33 ± 8.38 ^b^	51.33 ± 3.40 ^b^	44.67 ± 6.55 ^b^	48.00 ± 3.56 ^b^	93.56 ± 0.03 ^a^
a*	12.33 ± 4.99 ^a^	17.33 ± 6.18 ^a^	12.67 ± 3.86 ^a^	16.67 ± 4.50 ^a^	19.00 ± 6.38 ^a^	−18.42 ± 0.02 ^b^
b*	38.33 ± 8.73 ^b^	38.00 ± 19.03 ^b^	40.00 ± 13.95 ^b^	41.33 ± 15.69 ^b^	49.33 ± 5.44 ^b^	75.24 ± 0.08 ^a^
∆E*	62.66 ± 10.99 ^e^	69.97 ± 21.60 ^a^	63.17 ± 14.78 ^d^	69.07 ± 17.50 ^b^	64.40 ± 9.03 ^c^	0.00 ± 0.00 ^f^

1 (70%: 15%: 15%); 2 (60%: 25%: 15%); 3 (60%: 15%: 25%); 4 (50%: 25%: 25%); 5 (60%: 20%: 20%); 6 (Control: 100% date palm extract). Values are mean ± SD; n = 3 independent production batches × 2 analytical replicates. Different superscript letters within a row indicate significant differences (Tukey’s HSD, *p* ≤ 0.05). The letter “ns” indicates no statistically significant differences (*p* > 0.05) in each row.

#### 3.4.2. Functional Properties of Ready-to-Drink Beverage from Date Palm Optimized Extract

The principal advancement of this RTD beverage stems from the deliberate integration of three phytochemically distinct fruit matrices, which collectively amplify its functional attributes. All blended formulations exhibited statistically significant (*p* ≤ 0.05) elevations in bioactive compound concentrations relative to the 100% date palm control (Table 8). Specifically, TPC increased markedly from 364.67 mg GAE/100 mL in the control to a peak of 1291.33 mg GAE/100 mL in Formulation 4 (50% date palm: 25% bael: 25% jujube). Total carotenoid content (TCC) followed a parallel trajectory, rising from 1.09 to 7.55 mg β-CE/100 mL. These enhancements are ascribed to the complementary phytochemical architectures of bael and jujube: bael is recognized as a rich source of phenolic compounds, particularly chlorogenic acid, caffeoylquinic acids, and flavonols, whereas jujube is notably abundant in C-glycosyl flavonoids (e.g., spinosin, swertisin), immunomodulatory polysaccharides, and triterpenic acids such as betulinic acid [10,28]. When combined with the phenolic profile of date palm—which includes protocatechuic acid, ferulic acid, and β-carotene [2]—these constituents act synergistically to generate a beverage with a substantially enriched antioxidant matrix.

Correspondingly, in vitro antioxidant capacity, assessed via DPPH radical scavenging and FRAP assays, mirrored this upward trend. DPPH activity escalated from 525.52 µmol TE/100 mL (control) to 1535.13 µmol TE/100 mL (Formulation 4), while FRAP values increased from 603.95 to 1633.57 µmol TE/100 mL. These elevated metrics align with the observed increases in TPC and TCC, confirming that the strategic blending approach effectively amplified the beverage’s functional potency. Notably, the TPC (up to 1291.33 mg GAE/100 mL) and DPPH (1535.13 µmol TE/100 mL) values achieved in Formulation 4 are comparable to or exceed those reported for commercial pomegranate juices (≈900–1400 mg GAE/100 mL; DPPH ≈ 1100–1500 µmol TE/100 mL) and mixed-berry RTD products, and substantially surpass conventional single-fruit juices such as orange or apple (TPC ≈ 200–400 mg GAE/100 mL) [29,30]. These findings reinforce the concept that targeted multi-fruit blending represents a viable strategy for developing RTD beverages with superior antioxidant profiles that transcend the limitations of single-ingredient formulations.

Nevertheless, the stability of carotenoids and phenolics during processing and storage warrants careful consideration, as thermal exposure and oxidative conditions may diminish bioactivity over time [26,27]. Future stability studies will monitor the retention of key phytochemical markers under simulated commercial storage conditions to ensure functional integrity throughout the product’s shelf life.

### 3.5. Consumer Acceptance Tests

The commercial viability of any functional beverage ultimately hinges on consumer sensory acceptance. Hedonic testing conducted with 30 untrained panelists revealed statistically significant variations (*p* ≤ 0.05) across all evaluated attributes (Table 9). Notably, sensory preference did not align with the formulation exhibiting the highest antioxidant capacity. Although Formulation 4 (50% date palm: 25% bael: 25% jujube) maximized functional properties, Formulation 1 (70:15:15) achieved the highest overall acceptability score (6.50/9), corresponding to a “slight like.” This outcome suggests that a dominant date palm base, characterized by its familiar natural sweetness, provided a more palatable foundation that was effectively complemented by moderate additions of bael and jujube. Nevertheless, scores within the “moderate acceptance” range highlight potential sensory limitations. The elevated polyphenol load from bael and jujube may impart mild astringency or a bitter aftertaste, while the inherent viscosity of date palm extract could contribute to a heavier mouthfeel [26]. To improve consumer appeal, subsequent formulations could integrate natural acidulants (e.g., citric or malic acid) to enhance brightness, apply enzymatic clarification to minimize turbidity, introduce light carbonation for increased refreshment, or incorporate natural flavor modulators (e.g., citrus peel or mint extracts) to counteract polyphenolic bitterness without increasing sugar content. Additionally, fine-tuning the fruit-to-water infusion ratios for bael and jujube could further optimize the sweet-tart equilibrium for broader demographic targeting.

Jujube has historically been utilized in traditional medicine to promote stress resilience and support hormonal balance in women, effects largely attributed to its saponin and flavonoid constituents [10,28]. However, contemporary regulatory frameworks (e.g., FDA, EFSA) mandate rigorous clinical validation before any therapeutic or structure-function claims can be legally marketed [25]. Elevating the jujube proportion beyond 25% risks disrupting the established sweet-acid profile, increasing product viscosity, and potentially triggering herb-drug interactions in consumers undergoing hormonal treatments. Consequently, while future niche “wellness-oriented” products may explore optimized jujube dosages through targeted clinical trials, the present 70:15:15 formulation strategically balances broad consumer palatability, functional efficacy, and regulatory compliance. Definitive safety and efficacy assertions will require dedicated toxicological assessments and human intervention studies.

In contrast, the control sample (100% date palm extract) recorded the lowest overall preference (5.87) and the poorest color acceptance (5.54), indicating that despite its nutritional value, the unblended juice’s monotonous flavor and appearance were less attractive to consumers. The blended formulations consistently outperformed the control in color (up to 7.13) and aroma (up to 6.66), demonstrating that the visual and olfactory complexity introduced by bael and jujube was well-received. Sweetness and sourness ratings remained within a narrow, balanced range (5.00–6.20 and 5.22–6.00, respectively), confirming that no single taste attribute overwhelmed the others. This inverse relationship between peak functional potency (Formulation 4) and peak sensory appeal (Formulation 1) exemplifies a well-documented challenge in functional food design [31]. Formulation 1 emerges as a practical compromise, delivering substantially improved antioxidant capacity (TPC: 1023 mg GAE/100 mL; DPPH: 1394.79 µmol TE/100 mL) relative to the control while securing the highest hedonic ratings. From an industrial perspective, commercial scalability will be influenced by raw material sourcing and enzyme costs, while long-term product stability will necessitate proactive management of sedimentation and enzymatic browning [26,27].

Although the current consumer test effectively identified primary acceptability drivers, it did not capture the full aromatic complexity of the beverage. Volatile compounds characteristic of bael (e.g., limonene, benzaldehyde) and jujube (e.g., benzaldehyde, 2-phenylethanol) [9,10] likely played a significant role in shaping the observed aroma scores. Future investigations should integrate gas chromatography–mass spectrometry (GC-MS) with trained-panel descriptive analysis to systematically map aroma-taste interactions, thereby enabling precise flavor optimization and enhancing overall product refinement.

## 4. Conclusions

This study demonstrates that pectinase-assisted extraction (0.1% *v*/*v*, 50 °C, 60 min) optimally preserves heat-sensitive bioactives in date palm juice, yielding high total phenolic (326.33 mg GAE/100 mL) and carotenoid (1.08 mg β-CE/100 mL) contents alongside robust antioxidant activity (DPPH: 514.06; FRAP: 595.38 µmol TE/100 mL). When blended with bael and jujube juices using a constrained mixture design, the resulting ready-to-drink (RTD) formulations exhibited significantly enhanced functional properties—reaching up to 1291.33 mg GAE/100 mL TPC and 1535.13 µmol TE/100 mL DPPH—while maintaining stable pH and titratable acidity. Sensory evaluation identified the 70:15:15 (date palm: bael: jujube) blend as the most acceptable formulation (overall liking: 6.50/9), effectively balancing antioxidant potency with consumer preference. These findings confirm the technical feasibility of this tri-fruit matrix as a novel, health-promoting RTD beverage. While extraction and formulation parameters were successfully optimized, commercial translation requires comprehensive stability and safety validation. Future work will focus on microbial risk assessment, accelerated and real-time shelf-life trials, and processing interventions (e.g., pasteurization or high-pressure processing) to mitigate enzymatic browning, sedimentation, and bioactive degradation. Additionally, advanced volatile profiling (GC-MS) and targeted flavonoid quantification (HPLC-DAD/MS) will be implemented to refine flavor balance, verify functional retention during storage, and ensure regulatory compliance for market-ready functional beverages.

## Figures and Tables

**Table 1:** Extraction conditions of date palm.

	Extraction Conditions		
Order	Enzyme Concentration (%)	Temperature (°C)	Time (min)
1	0	50	60
2	0	60	120
3	0	70	180
4	0	80	240
5	0.1	50	60
6	0.1	60	120
7	0.1	70	180
8	0.1	80	240

**Table 2:** Proportions of date palm juice with bael juice and fountain for a ready-to-drink beverage.

Formulation	Date Palm Extract (%)	Bael Juice (%)	Jujube Juice (%)	Description
1	70	15	15	70:15:15
2	60	25	15	60:25:15
3	60	15	25	60:15:25
4	50	25	25	50:25:25
5	60	20	20	60:20:20
Control	100	0	0	100% date palm extract

**Table 3:** Effect of pectinase-assisted extraction conditions of date palm on the extraction yield (%).

Treatments								
Time (min)	Control (No Enzyme)				Pectinase Enzyme (0.1 *v*/*v*)			
	50 °C	60 °C	70 °C	80 °C	50 °C	60 °C	70 °C	80 °C
60	58.49 ± 6.16 ^B, b^	59.03 ± 0.98 ^B, b^	65.96 ± 5.54 ^A, a^	61.49 ± 5.33 ^A, ab^	65.94 ± 4.47 ^B, b^	69.44 ± 5.20 ^B^^,^^b^	77.81 ± 7.91 ^A, a^	66.09 ± 2.15 ^A, b^
120	62.26 ± 1.54 ^AB, ab^	61.81 ± 1.96 ^B, b^	64.48 ± 3.91 ^A, a^	60.92 ± 2.15 ^A, b^	68.84 ± 2.71 ^B, b^	72.92 ± 6.13 ^B, a^	73.75 ± 6.16 ^A, a^	64.94 ± 2.93 A^B, b^
180	67.92 ± 1.54 ^A, a^	66.67 ± 1.70 ^AB, a^	57.22 ± 4.48 ^B, b^	56.90 ± 1.41 ^B, b^	75.36 ± 6.23 ^AB, a^	78.47 ± 2.60 ^AB, a^	71.30 ± 2.37 ^AB, a^	64.94 ± 3.54 ^AB, b^
240	69.81 ± 2.67 ^A, a^	70.14 ± 4.91 ^A, a^	56.76 ± 1.03 ^B, b^	54.60 ± 2.15 ^B, b^	80.43 ± 1.77 ^A, a^	81.25 ± 1.70 ^A, a^	67.22 ± 5.96 ^B, b^	63.22 ± 3.54 ^B, b^

The results are presented as Mean ± Standard Deviation. Different uppercase letters (A,B) within a column indicate significant differences among time points at the same temperature and enzyme level (Tukey’s HSD, *p* ≤ 0.05). Different lowercase letters (a,b) within a row indicate significant differences among temperatures at the same time.

**Table 4:** Effect of pectinase-assisted extraction conditions of date palm on total soluble solid content (°Brix).

	Treatments							
Time (min)	Control				Pectinase Enzyme 0.1% *v*/*v*			
	50 °C ^ns^	60 °C ^ns^	70 °C ^ns^	80 °C ^ns^	50 °C ^ns^	60 °C ^ns^	70 °C ^ns^	80 °C ^ns^
60 ^NS^	6.5 ± 0.08	6.5 ± 0.05	6.7 ± 0.08	6.6 ± 0.05	6.6 ± 0.08	6.6 ± 0.05	6.7 ± 0.05	6.7 ± 0.08
120 ^NS^	6.6 ± 0.14	6.5 ± 0.05	6.5 ± 0.12	6.5 ± 0.12	6.6 ± 0.12	6.7 ± 0.08	6.7 ± 0.08	6.6 ± 0.05
180 ^NS^	6.6 ± 0.08	6.5 ± 0.05	6.5 ± 0.09	6.5 ± 0.08	6.7 ± 0.16	6.7 ± 0.05	6.6 ± 0.05	6.6 ± 0.08
240 ^NS^	6.7 ± 0.16	6.7 ± 0.05	6.5 ± 0.08	6.4 ± 0.05	6.7 ± 0.12	6.8 ± 0.08	6.6 ± 0.08	6.5 ± 0.09

Mean ± Standard Deviation. The superscript “ns” means that the average of each column has no statistically significant difference (*p* > 0.05). The superscript “NS” means that the average of each row has no statistically significant difference (*p* > 0.05).

**Table 5:** Effect of pectinase-assisted extraction conditions of date palm on color values.

	Treatments								
	Time (min)	Control (No Enzyme)				Pectinase Enzyme (0.1% *v*/*v*)			
		50 °C	60 °C	70 °C	80 °C	50 °C	60 °C	70 °C	80 °C
L*	60	91.13 ± 0.05 ^B, a^	90.50 ± 0.04 ^B, a^	85.44 ± 0.03 ^A, b^	79.50 ± 0.38 ^A, c^	93.56 ± 0.30 ^B, a^	92.96 ± 0.02 ^B, a^	86.87 ± 0.04 ^A, b^	84.61 ± 0.20 ^A, b^
	120	91.32 ± 0.04 ^B, a^	90.58 ± 0.16 ^B, a^	85.16 ± 0.06 ^A, b^	79.24 ± 0.17 ^A, c^	93.91 ± 0.06 ^AB, a^	93.16 ± 0.03 ^AB, a^	86.68 ± 0.17 ^A, b^	84.34 ± 0.24 ^A, b^
	180	91.66 ± 0.07 ^AB, a^	91.62 ± 0.01 ^A, a^	84.72 ± 0.02 ^B, b^	78.91 ± 0.43 ^AB, c^	94.21 ± 0.03 ^A, a^	93.24 ± 0.0 ^A, a^	85.96 ± 0.02 ^AB, b^	83.30 ± 0.12 ^B, b^
	240	92.13 ± 0.05 ^A, a^	91.08 ± 0.03 ^A, a^	84.61 ± 0.11 ^B, b^	78.78 ± 0.39 ^B, c^	94.58 ± 0.02 ^A, a^	93.49 ± 0.02 ^A, a^	85.85 ± 0.02 ^B, b^	83.26 ± 0.06 ^B, b^
a*	60	−16.70 ± 0.0 ^NS, b^	−20.17 ± 0.01 ^NS, c^	−17.41 ± 0.20 ^B, b^	−14.32 ± 0.40 ^A, a^	−18.42 ± 0.02 ^A, a^	−20.19 ± 0.02 ^AB, b^	−19.83 ± 0.01 ^NS^	−18.57 ± 0.34 ^C, a^
	120	−16.69 ± 0.03 ^NS, a^	−20.71 ± 0.01 ^NS, c^	−17.77 ± 0.05 ^B, b^	−16.50 ± 0.27 ^B, a^	−18.61 ± 0.01 ^A, ab^	−20.92 ± 0.01 ^A, c^	−19.17 ± 0.10 ^NS, b^	−17.57 ± 0.33 ^B, a^
	180	−16.65 ± 0.06 ^NS, a^	−20.55 ± 0.04 ^NS, c^	−16.12 ± 0.08 ^A, a^	−18.45 ± 0.06 ^C, b^	−21.49 ± 0.02 ^B, c^	−21.03 ± 0.01 ^B, c^	−19.16 ± 0.04 ^NS, b^	−16.47 ± 0.36 ^A, a^
	240	−16.58 ± 0.03 ^NS, a^	−20.80 ± 0.01 ^NS, b^	−17.98 ± 0.01 ^AB, a^	−16.36 ± 0.13 ^B, a^	−21.49 ± 0.04 ^B, b^	−21.16 ± 0.05 ^B, b^	−19.15 ± 0.03 ^NS, a^	−21.44 ± 0.09 ^D, b^
b*	60	64.64 ± 0.37 ^NS, c^	84.16 ± 0.01 ^NS, a^	73.25 ± 0.01 ^A, b^	48.23 ± 0.07 ^D, d^	75.24 ± 0.08 ^B, b^	84.94 ± 0.04 ^NS, a^	58.71 ± 0.04 ^NS, d^	61.23 ± 0.09 ^AB, c^
	120	64.63 ± 0.30 ^NS, c^	84.52 ± 0.04 ^NS, a^	70.37 ± 0.07 ^B, b^	56.39 ± 0.03 ^C, d^	75.70 ± 0.09 ^B, b^	84.83 ± 0.05 ^NS, a^	58.81 ± 0.02 ^NS, c^	57.17 ± 0.02 ^B, c^
	180	64.62 ± 0.29 ^NS, b^	84.57 ± 0.04 ^NS, a^	57.43 ± 0.02 ^C, c^	61.49 ± 0.04 ^B, b^	82.31 ± 0.01 ^A, a^	84.98 ± 0.03 ^NS, a^	58.84 ± 0.02 ^NS, c^	66.31 ± 0.06 ^A, b^
	240	64.71 ± 0.05 ^NS, c^	84.44 ± 0.02 ^NS, a^	71.30 ± 0.05 ^B, b^	66.52 ± 0.03 ^A, c^	82.42 ± 0.06 ^A, a^	84.97 ± 0.03 ^NS, a^	58.94 ± 0.03 ^NS, b^	56.31 ± 0.04 ^B, b^
∆E*	60	0.00 ± 0.00	1983 ± 0.36 ^B, a^	10.35 ± 0.40 ^A, b^	20.26 ± 0.59 ^A, a^	11.01 ± 0.29 ^B, b^	20.68 ± 0.33 ^B, a^	7.95 ± 0.33 ^AB, c^	7.59 ± 0.45 ^C, c^
	120	0.20 ± 0.07 ^B, d^	20.29 ± 0.34 ^A, a^	8.34 ± 0.30 ^C, c^	14.47 ± 0.44 ^B, b^	11.57 ± 0.28 ^B, b^	20.73 ± 0.32 ^B, a^	7.73 ± 0.38 ^B, c^	10.13 ± 0.51 ^B, b^
	180	0.53 ± 0.09 ^B, d^	2030 ± 0.34 ^A, a^	9.66 ± 0.35 ^B, c^	1274 ± 0.50 ^C, b^	18.57 ± 0.36 ^A, b^	20.90 ± 0.34 ^A, a^	8.15 ± 0.35 ^A, c^	8.01 ± 0.47 ^C, c^
	240	1.02 ± 0.32 ^A, d^	20.22 ± 0.35 ^A, a^	941 ± 0.33 ^B, c^	12.49 ± 0.49 ^C, b^	1873 ± 0.31 ^A, b^	20.95 ± 0.34 ^A, a^	8.14 ± 0.34 ^A, c^	12.40 ± 0.34 ^A, bc^

Mean ± Standard Deviation. Different letters (A–D) refer to the significant differences (*p* ≤ 0.05) in each column. NS: non-significant in each column. Different letters (a–d) refer to the significant differences (*p* ≤ 0.05) in each row.

**Table 6:** Effect of pectinase-assisted extraction conditions of date palm on functional properties.

	Treatments								
	Time (min)	Control Samples				Pectinase Enzyme 0.1% *v*/*v*			
		50 °C	60 °C	70 °C	80 °C	50 °C	60 °C	70 °C	80 °C
TPC	60	313.00 ± 22.73 ^A, a^	271.33 ± 22.48 ^A, b^	258.00 ± 8.16 ^A, c^	199.67 ± 10.27 ^A, d^	326.33 ± 23.21 ^A, a^	288.00 ± 14.72 ^A, b^	243.00 ± 32.66 ^A, c^	178.00 ± 16.33 ^A, d^
	120	303.00 ± 21.60 ^AB, a^	269.67 ± 12.47 ^AB, b^	241.33 ± 4.71 ^AB, c^	178.00 ± 16.33 ^AB, d^	318.00 ± 14.72 ^AB, a^	273.00 ± 10.80 ^AB, b^	231.33 ± 13.12 ^AB, c^	161.33 ± 20.95 ^AB, d^
	180	291.33 ± 18.41 ^BC, a^	261.33 ± 10.27 ^BC, b^	234.67 ± 10.27 ^BC, c^	154.67 ± 8.50 ^BC, d^	303.00 ± 24.49 ^BC, a^	263.00 ± 18.71 ^BC, b^	221.33 ± 6.24 ^BC, c^	148.00 ± 10.80 ^BC, d^
	240	281.33 ± 24.94 ^C, a^	259.67 ± 16.50 ^C, b^	229.67 ± 13.12 ^C, c^	144.67 ± 12.47 ^C, d^	286.33 ± 10.27 ^C, a^	261.33 ± 28.96 ^C, b^	194.67 ± 6.24 ^C, c^	134.67 ± 14.34 ^C, d^
TCC	60	1.05 ± 0.05 ^A, a^	0.74 ± 0.02 ^A, b^	0.56 ± 0.04 ^A, c^	0.54 ± 0.02 ^A, c^	1.08 ± 0.04 ^A, a^	0.76 ± 0.02 ^A, b^	0.62 ± 0.04 ^A, c^	0.59 ± 0.02 ^A, c^
	120	0.99 ± 0.12 ^A, a^	0.70 ± 0.06 ^A, b^	0.53 ± 0.01 ^A, c^	0.50 ± 0.04 ^A, c^	1.05 ± 0.04 ^A, a^	0.72 ± 0.02 ^A, b^	0.56 ± 0.05 ^B, c^	0.55 ± 0.05 ^A, c^
	180	0.96 ± 0.02 ^B, a^	0.62 ± 0.02 ^B, b^	0.44 ± 0.04 ^B, c^	0.39 ± 0.03 ^B, c^	0.99 ± 0.03 ^AB, a^	0.66 ± 0.01 ^B, b^	0.49 ± 0.04 ^C, c^	0.46 ± 0.04 ^B, c^
	240	0.90 ± 0.06 ^C, a^	0.54 ± 0.09 ^C, b^	0.33 ± 0.06 ^C, c^	0.33 ± 0.12 ^B, c^	0.96 ± 0.07 ^B, a^	0.58 ± 0.04 ^C, b^	0.48 ± 0.04 ^C, c^	0.41 ± 0.02 ^C, c^
DPPH	60	495.73 ± 14.36 ^A, a^	413.23 ± 30.15 ^A, b^	374.48 ± 4.57 ^A, c^	323.65 ± 21.30 ^A, d^	514.06 ± 17.94 ^A, a^	426.77 ± 17.10 ^A, b^	359.90 ± 14.77 ^A, c^	294.06 ± 28.74 ^A, d^
	120	479.06 ± 35.59 ^AB, a^	405.10 ± 13.78 ^AB, b^	347.40 ± 1.06 ^AB, c^	318.85 ± 11.64 ^AB, d^	513.65 ± 11.09 ^A, a^	424.48 ± 3.90 ^A, b^	338.23 ± 13.59 ^AB, c^	273.02 ± 14.74 ^AB, d^
	180	470.52 ± 38.81 ^B, a^	401.98 ± 15.53 ^AB, b^	332.81 ± 20.10 ^B, c^	305.52 ± 16.16 ^B, d^	496.35 ± 24.44 ^B, a^	420.31 ± 7.14 ^AB, b^	311.35 ± 33.82 ^B, c^	265.31 ± 12.79 ^B, d^
	240	415.94 ± 11.37 ^C, a^	392.40 ± 4.40 ^B, b^	324.48 ± 21.62 ^C, c^	284.90 ± 36.55 ^C, d^	452.40 ± 16.33 ^C, a^	411.15 ± 7.91 ^B, b^	291.35 ± 19.14 ^C, c^	243.23 ± 16.66 ^C, d^
FRAP	60	565.50 ± 23.18 ^A, a^	497.17 ± 16.48 ^A, b^	389.31 ± 13.25 ^A, c^	312.05 ± 5.22 ^A, d^	595.38 ± 12.38 ^A, a^	519.55 ± 7.75 ^A, b^	358.00 ± 4.40 ^A, c^	291.81 ± 2.48 ^A, d^
	120	558.36 ± 7.00 ^AB, a^	487.17 ± 25.75 ^AB, b^	385.98 ± 51.25 ^A, c^	298.95 ± 15.24 ^AB, d^	569.43 ± 12.96 ^AB, a^	509.67 ± 31.43 ^AB, b^	339.19 ± 12.70 ^AB, c^	271.10 ± 19.80 ^AB, d^
	180	539.07 ± 21.58 ^BC, a^	477.17 ± 43.91 ^BC, b^	374.79 ± 9.45 ^BB, c^	273.36 ± 17.56 ^BC, d^	553.83 ± 20.11 ^BC, a^	490.74 ± 13.07 ^BC, b^	323.83 ± 46.39 ^BC, c^	253.00 ± 23.70 ^BC, d^
	240	501.33 ± 20.61 ^C, a^	466.69 ± 5.84 ^C, b^	352.64 ± 41.28 ^C, c^	268.24 ± 16.26 ^C, d^	520.38 ± 6.74 ^C, a^	477.64 ± 28.80 ^C, b^	308.60 ± 33.82 ^C, c^	240.98 ± 34.49 ^C, d^

Different letters (A–C) refer to the significantly differences (*p* ≤ 0.05) in each column. Different letters (a–d) refer to the significantly differences (*p* ≤ 0.05) in each row. TPC = Total phenolic content, expressed as mg gallic acid equivalents (GAE)/100 mL. TCC = Total carotenoid content, expressed as mg β-carotene equivalents/100 mL. DPPH and FRAP = Antioxidant activity, expressed as µmol Trolox equivalents (TE)/100 mL.

**Table 8:** Effects of optimized pectinase-assisted extraction of date palm on functional characteristics of Ready-to-Drink beverages.

	Treatments (Date Palm Juice Extract: Bael Juice: Jujube Juice)					
	1	2	3	4	5	6
TPC	1023.00 ± 26.77 ^b^	1206.33 ± 12.47 ^a^	973.00 ± 10.80 ^b^	1291.33 ± 41.70 ^a^	994.67 ± 31.18 ^b^	364.67 ± 17.00 ^c^
TCC	7.02 ± 0.07.00 ^a^	7.06 ± 1.05.00 ^a^	5.56 ± 0.25.00 ^b^	7.55 ± 0.51.00 ^a^	6.18 ± 0.04.00 ^b^	1.09 ± 0.10.00 ^c^
DPPH	1394.79 ± 36.92 ^bc^	1530.21 ± 38.30 ^a^	1211.46 ± 60.88 ^c^	1535.13 ± 36.80 ^a^	1505.00 ± 15.59	525.52 ± 27.99 ^d^
FRAP	1487.14 ± 10.51 ^bc^	1600.24 ± 37.15	1289.52 ± 66.52 ^d^	1633.57 ± 15.43 ^a^	1394.29 ± 45.27 ^cd^	603.95 ± 50.36 ^e^

1 (70%: 15%: 15%); 2 (60%: 25%: 15%); 3 (60%: 15%: 25%); 4 (50%: 25%: 25%); 5 (60%: 20%: 20%); 6 (Sample Control). Values are mean ± SD; n = 3 independent production batches × 2 analytical replicates. Different superscript letters within a row indicate significant differences (Tukey’s HSD, *p* ≤ 0.05). TPC = Total phenolic content, expressed as mg gallic acid equivalents (GAE)/100 mL TCC = Total carotenoid content, expressed as mg β-carotene equivalents/100 mL. DPPH and FRAP = Antioxidant activity, expressed as µmol Trolox equivalents (TE)/100 mL.

**Table 9:** Effects of optimized pectinase-assisted extraction of date palm on sensory evaluations of Ready-to-Drink beverages.

	Treatments (Date Palm Juice Extract: Bael Juice: Jujube Juice)					
	1	2	3	4	5	6
Color	6.92 ± 1.44 ^a^	7.13 ± 1.23 ^a^	7.12 ± 1.09 ^a^	7.13 ± 1.27 ^a^	6.62 ± 1.11 ^a^	5.54 ± 1.99 ^b^
Smell	6.51 ± 1.45 ^a^	6.66 ± 1.18 ^a^	6.41 ± 1.61 ^a^	6.14 ± 1.35 ^a^	6.38 ± 1.56 ^a^	5.77 ± 1.86 ^b^
Sweetness	6.20 ± 1.55 ^a^	5.83 ± 1.56 ^a^	5.66 ± 1.75 ^a^	5.00 ± 1.60 ^b^	5.94 ± 1.54 ^a^	5.91 ± 1.89 ^a^
Sour taste	6.00 ± 1.82 ^a^	5.70 ± 1.62 ^b^	5.83 ± 1.80 ^b^	5.22 ± 1.63 ^b^	5.96 ± 1.68 ^b^	5.53 ± 2.04 ^b^
Flavor	6.24 ± 1.61 ^a^	5.98 ± 1.52 ^b^	5.98 ± 1.83 ^b^	5.59 ± 1.70 ^b^	5.96 ± 1.81 ^b^	6.21 ± 2.00 ^a^
Overall Preferences	6.50 ± 1.61 ^a^	5.97 ± 1.56 ^b^	6.00 ± 1.68 ^b^	5.41 ± 1.55 ^b^	5.96 ± 1.64 ^b^	5.87 ± 2.15 ^b^

1 (70%: 15%: 15%); 2 (60%: 25%: 15%); 3 (60%: 15%: 25%); 4 (50%: 25%: 25%); 5 (60%: 20%: 20%); 6 (Control:100% date palm extract). Values are mean ± SD; n = 30. Different superscript letters within a row indicate significant differences (Tukey’s HSD, *p* ≤ 0.05).

## Data Availability

The original contributions presented in this study are included in the article. Further inquiries can be directed to the corresponding author.
