# Correction: Jafari et al. Optimization of Pectinase-Assisted Extraction from Date Palm and Development of a Bael–Jujube Ready-to-Drink Beverage: A Two-Stage Approach. *Foods* 2026, *15*, 1394

**DOI:** 10.3390/foods15132309

**Published:** 2026-06-29

**Authors:** Saeid Jafari, Pitchaya Tuntiteeraboon, Isaya Kijpatanasilp, Sochannet Chheng, Kuan-Chen Cheng, Kitipong Assatarakul

**Affiliations:** 1 Department of Food Technology, Faculty of Science, Chulalongkorn University, Bangkok 10330, Thailand; saeid.j@chula.ac.th (S.J.); ykijpat@gmail.com (I.K.); sochannetc@gmail.com (S.C.); 2 Department of Food Chemical Engineering, Kampong Speu Institute of Technology, Chbar Mon 050601, Cambodia; 3 Institute of Food Science and Technology, National Taiwan University, No. 1, Section 4, Roosevelt Rd., Taipei 106216, Taiwan; kccheng@ntu.edu.tw; 4 Institute of Biotechnology, National Taiwan University, No. 1, Section 4, Roosevelt Rd., Taipei 106216, Taiwan; 5 Department of Optometry, Asia University, No. 500, Lioufeng Rd., Wufeng, Taichung 413305, Taiwan; 6 Department of Medical Research, China Medical University Hospital, China Medical University, No. 91, Hsueh-Shih Road, Taichung 404333, Taiwan; 7 Department of Food Science, Fu Jen Catholic University, No. 510, Zhongzheng Rd., New Taipei City 242062, Taiwan

## Text Correction

There were errors in the original publication [1], the misuse of the English term “Bael” in the whole article.

A correction has been made to the word “Quince” to “Bael” in the whole article, with corresponding adjustments to the following affected sentences and reference [9].

In Section 1 paragraph 3, the correction has been made: “Bael juice contains carotenoids, phenolics, alkaloids, pectins, tannins, coumarins, flavonoids and terpenoids. In the Ayurvedic system of medicine, it can be use to aid digestion and stomach irritation and prevent sub-acute and chronic dysentery due to its biological properties [9].”

In Section 1 paragraph 4, the correction has been made: “bael contributes moderate acidity and is a good source of pectin; it also contains phenolic compounds, including chlorogenic acid, which contribute to its antioxidant capacity without imparting excessive tartness, while jujube introduces unique neuroprotective flavonoids (spinosin, swertisin) and a subtle, floral-sweet aroma that rounds out the flavor profile [9].”

In Section 3.4.2 paragraph 1, the correction has been made: “bael is recognized as a rich source of phenolic compounds, particularly chlorogenic acid, caffeoylquinic acids, and flavonols”.

In Section 3.5 paragraph 4, the correction has been made: “Volatile compounds characteristic of bael (e.g., limonene, benzaldehyde)”.

9. Baliga, M.S.; Bhat, H.P.; Joseph, N.; Fazal, F. Phytochemistry and medicinal uses of the bael fruit (*Aegle marmelos* Correa): A concise review. *Food Res. Int.* **2011**, *44*, 1768–1775.

The authors state that the scientific conclusions are unaffected. This correction was approved by the Academic Editor. The original publication has also been updated.
